# From Retrograde Menstruation to Endometrial Determinism and a Brave New World of “Root Treatment” of Endometriosis: Destiny or a Fanciful Utopia?

**DOI:** 10.3390/biom13020336

**Published:** 2023-02-09

**Authors:** Sun-Wei Guo, Marwan Habiba, Giuseppe Benagiano

**Affiliations:** 1 Research Institute, Shanghai Obstetrics & Gynecology Hospital, Fudan University, Shanghai 200011, China; 2 Department of Health Sciences, University of Leicester, Leicester LE1 7HA, Leicestershire, UK; 3 Women and Perinatal Services, Leicester Royal Infirmary, Leicester LE1 5WW, Leicestershire, UK; 4 Faculty of Medicine and Dentistry, Sapienza, University of Rome, 00185 Roma, Italy

**Keywords:** aberration, causal, endometriosis, endometrial determinism, phylogenetic, retrograde menstruation, root treatment

## Abstract

Practically unknown outside of China, the “endometrial determinism” theory was proposed to account for the apparent gap between the relatively low prevalence of endometriosis and nearly universal retrograde menstruation. Attracting uncritical advocacy, the theory culminates in a recent consensus by elite Chinese gynecologists in favor of “root treatment”, intended to nip endometriosis in the bud. Correcting endometrial “defects” can gain further momentum by the presence of cancer-driver mutations such as KRAS mutations in the endometrium of women with endometriosis and the recent introduction of therapeutics aiming to rectify the effect of these mutations for cancer treatment. We provide a critical appraisal of evidence for endometrial aberrations in endometriosis and relevant experimental evidence. All available evidence of endometrial “defect” is invariably post hoc and may well be secondary to induced endometriosis. We propose that the theory of “endometrial determinism” needs to demonstrate a clear causal and a phylogenetic relationship between endometrial aberrations and endometriosis. We argue that while it is highly likely that endometriosis is a consequence of retrograde menstruation, the case that molecular aberrations as a sole or a necessary determinant remains to be proven. “Root treatment” is a worthy ambition but as of now it is close to a fanciful Utopia.

## 1. Introduction

Among all theories on the pathogenesis of endometriosis, Sampson’s retrograde menstruation has the greatest supporting evidence. Written nearly a century ago, Sampson proposed that “retrograde transport of menstrual blood with the consequent implantation of exfoliated endometrial mucosa cells within the peritoneal cavity” causes what he named “endometriosis” [1]. The validity of this theory was first demonstrated in 1950 in a *Macacus rhesus* monkey experimentation by the successful establishment of endometriosis through artificial shunting of menstrual debris into the abdominal cavity [2] and, in 1958, through subcutaneous inoculation at “one finger breadth above the symphysis pubis in the midline” in humans [3]. Later on, it was shown that intrapelvic injection of menstrual endometrium in female baboons can induce endometriosis [4]. The baboon model was further modified by peritoneal inoculation, twice, with menstrual endometrial debris leading to the establishment of endometriotic foci [5]. In addition, there is a well-documented link between reproductive tract obstruction that enhances retrograde menstrual flux and endometriosis [2]. Thus, experimental data, as well as epidemiological findings, support Sampson’s retrograde menstruation theory.

However, among women with patent fallopian tubes, retrograde menstruation is nearly universal [6], whereas the prevalence of endometriosis is typically around 10% [7]. Hence the conundrum: If retrograde menstruation is ubiquitous and nearly universal, why is there such a vast gap in prevalence? In fact, the 10% prevalence refers to all women of reproductive age. If we consider the age-specific prevalence, the gap would be even more glaring.

A comprehensive review, published over two decades ago, tallied several explanations for this apparent gap [8]. It elaborated the steps required for the establishment of endometriotic lesions through retrograde menstruation, i.e., reflux, adhesion, proteolysis, proliferation, angiogenesis, and cicatrization [8]. The possibility was raised that abnormal endometrium is the foremost predisposing factor for endometriosis. Specifically, the endometrium should have the ability to evade immune detection either through the possession of antigenic capability, harboring immune cell populations different from those of healthy women, and the synthesis and release of immune regulator; produce estrogens in excess and enhance survival, invasive and implantation propensity; reduce apoptotic tendency, carry molecules harmful to the peritoneum, or enhance angiogenic capability [8]. In other words, it is the seed, i.e., the defective endometrium, that is chiefly responsible for endometriosis. 

In China, this hypothesis went much further and was elevated to a full-fledged theory, termed “endometrial determinism”, which “revamped and refined Sampson’s hypothesis” [9]. Practically unknown outside of China, but acclaimed by many as the maestro stroke in the most populous nation with likely the highest number of women with endometriosis in the world, the theory claims to be able to perfectly explain the huge gap between nearly universal retrograde menstruation and the 10% prevalence of the disease [9]. In essence, the theory postulates that the occurrence of endometriosis is mainly dependent on the characteristics of the eutopic endometrium and that retrograde menstruation may act merely as the precipitating factor [9]. In particular, endometriosis originates from eutopic endometrium through a process of “3A” (namely, attachment, aggression and angiogenesis) [10,11] and, as such, treatment should start from its root causes, or “root treatment”, aimed at rectifying the endometrium and preventing endometriosis [12]. Winning accolades and numerous awards, the “endometrial determinism” theory has gained a firm traction in gynecology in China, attracting a huge crowd of cult-like followers. Based on this theory, a recent consensus opinion by a group of elite Chinese gynecologists, published in the flagship obstetrics/gynecology journal in China, proposed that endometriosis should be treated “from its root cause”, i.e., the abnormal endometrium [13]. 

The credence of defective endometrium responsible for endometriosis seems to have been recently bolstered by sequencing data demonstrating increased cancer-associated mutations (CAMs) in both eutopic and ectopic endometrium, implicating that the retrograde flow of CAM-harboring endometrial cells will confer selective advantages at ectopic sites that may lead to the development of endometriosis [14]. This implies that if those women whose CAMs-carrying endometrium can be detected early and certain counter measures are taken, endometriosis could be prevented.

Indeed, one specific CAM, i.e., the KRAS (G12C) mutation, which occurs in endometrium of some women with endometriosis [14], has been shown to be linked with the reduced expression of progesterone receptors (PGR) in endometrial epithelial cells, conferring resistance to progestin treatment [15]. The idea of rectifying the endometrium can be further buoyed and emboldened by the recent US Food and Drug Administration approval of Sotorasib, a first-in-class specific small molecule that irreversibly inhibits KRAS (G12C) [16]. 

Thus, we could be at the dawn of a brave new world in which the “root treatment” would potentially forestall the genesis of endometriosis for good, sparing millions of women worldwide from pain and suffering, the dashed dream of having a family, endless distress, fear of uncertainty of surgery and recurrence, lost productivity, school absenteeism, strained relationships and emotional, social and financial tolls and alleviating enormous economic burden to the society. 

This rosy picture nonetheless raises a fundamental question: Is the “root treatment” ultimately the destiny of our endeavor or merely a fanciful Utopia? More specifically, do these claims have any merit or are they simply whimsical and wishful thinking, unsubstantiated by the existing data?

In this review, we tasked ourselves with three questions: First, are reported eutopic endometrial aberrations in women with endometriosis the cause or merely the consequence of endometriosis? Second, can the presence of CAMs in eutopic endometrium be taken as evidence that endometriosis originates from defective endometrium? Lastly, is the “root treatment” practical or biologically plausible? Since the origin of endometriosis is of such fundamental importance to our understanding of its pathogenesis and possible prevention, in this review, we will weigh the evidence for and against the notion that endometriosis originates from aberrant endometrium. 

## 2. Aberrations in Eutopic Endometrium: Cause or Consequence of Endometriosis?

The presence of various molecular and cellular aberrations in the endometrium of women with endometriosis has been extensively reported. Searching PubMed using the phrase “endometriosis and (endometrial or endometrium)“ yielded 7109 papers (accessed on 1 February 2023), published since 2000 when aberrations reported before were comprehensively reviewed by Vantier et al. [8]. Indeed, expression profiling studies typically report tens or hundreds of genes, miRNAs, or long non-coding RNAs differentially expressed in the endometrium between women with endometriosis and without [17,18,19,20,21,22,23]. The evaluation of changes in DNA methylome yielded similar findings [24,25]. A comprehensive review, published in 2016, of published endometrial biomarkers of endometriosis involving 2729 participants concluded that only “17βHSD2, IL-1R2, caldesmon (a binding protein capable of regulating actomyosin contraction), and a number of neural markers (VIP, CGRP, SP, NPY and combination of VIP, PGP 9.5 and SP) showed promising evidence of diagnostic accuracy, but there was insufficient or poor quality evidence for any clinical recommendations.” [26].

Thus, considering the sheer number and type of aberrations, it may not be entirely informative to tally these aberrations, especially since different studies often yield conflicting results and because such differences vanish under refined grouping [27]. 

Instead, we opted to select a few notable, somewhat well known and important aberrations that span from “unable to confirm” to “consistently confirmed”. The first such aberration is the increased nerve fiber density in the endometrium. First reported in 2006 [28], it was subsequently consistently observed by the same research team [29,30], which went on to demonstrate, in a double-blinded study, an impressive specificity and sensitivity of 83% and 98%, respectively, in diagnosing endometriosis [31]. More remarkably, this finding was independently corroborated by a Belgian team, reporting a nearly perfect 95% sensitivity, 100% specificity and 97.5% accuracy in predicting the presence of minimal–mild endometriosis using just three neural markers [32].

Unfortunately, subsequent studies failed to replicate the relationship between endometriosis and increased nerve fiber density in the endometrium. Instead, they found that endometrial innervation is pain-dependent, rather than endometriosis specific [33,34]. Indeed, several later studies found either no such difference [35] or unacceptable sensitivity and specificity (32% and 46%, respectively, as reported in [36], and 64% and 50%, respectively, as reported in [37]). Consistent with the observation that the endometrial hyperinnervation is pain-, but not endometriosis-related, the estimates of sensitivity and specificity are the lowest when subjects who complained of pelvic pains were included [36]. 

**Figure 1:**
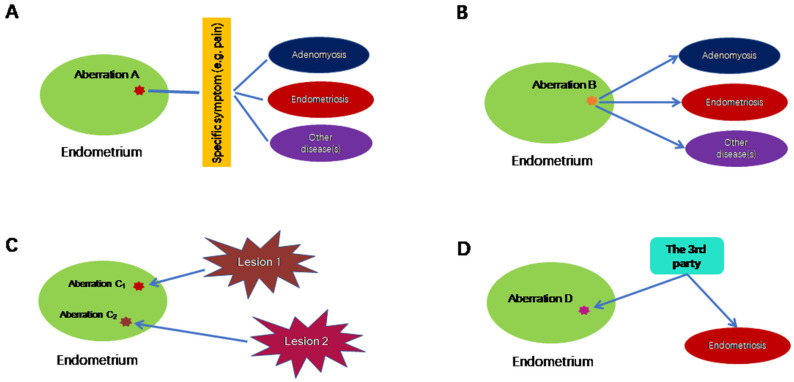
Possible scenarios in which endometrial aberrations do not cause endometriosis exclusively. (**A**) In this scenario, endometrial aberration A may be associated with a particular symptom, which is not specific to endometriosis. (**B**) Endometrial aberration B could lead to multiple conditions, including endometriosis. (**C**) Endometrial aberrations C_1_ and C_2_ are the consequences of endometriosis, where C_1_ and C_2_ are induced by different lesions of possibly different subtypes and/or their proximity to uterus. (**D**) Endometrial aberration D, along with endometriosis, is the result of a third, unknown factor. Lines without arrows indicate an association. The directional arrows indicate the causal relationship.

Thus, 16 years after its first report, endometrial hyperinnervation as quantitated by the nerve fiber density in endometrium has not become a diagnostic tool for endometriosis as of today. In essence, this example illustrates that an endometrial aberration may be linked with a non-specific symptom, such as dysmenorrhea or pelvic pain, which may be shared by many disorders, including endometriosis, but not endometriosis exclusively, as shown in Figure 1A. 

The second aberration is the gene or protein expression levels of PGRs. Progesterone is known to be a critical sex steroid hormone for the endometrium, and it also exerts a therapeutic effect on ectopic endometrium. Progesterone, as well as progestins, mediates their effects through two isoforms of PGR, namely PR-A and PR-B. Both PR-A and PR-B are encoded by the same gene, PGR, and, as such, both isoforms are identical in sequence, except PR-A is short of 164 amino acids at the N terminus, since both are transcribed from the same gene via two independent specific promoter regions and translation start sites [38]. Since progestins constitute an important class of drugs in the limited armamentarium of medical treatment of endometriosis, and since progesterone resistance (manifesting itself clinically as refractoriness to progestin treatment) is well-documented in endometriosis, PGR expression in both eutopic and ectopic endometrium is of major interest in endometriosis [39]. 

In endometriotic lesions, the reduced expression of both PR-A and PR-B, especially the latter, has been well-documented [40,41,42]. In fact, promoter hypermethylation of PR-B, which is responsible for its silence in endometriosis as well as in adenomyosis, has been reported as early as 2006 [43,44], and is likely caused by persistent inflammation [45]. Suppressed expression of PR-B in endometriosis apparently endows enhanced proliferative propensity [46]. 

In eutopic endometrium, the tendency for progesterone resistance has been also well-established [17,18]. Published data predominantly show PGR, especially PR-B, expression is either lost or reduced in the eutopic endometrium from women with endometriosis [42,47,48,49,50,51,52]. There is evidence for PR-B promoter hypermethylation, which may account for its loss [43,53]. However, negative reports also have been reported [54,55,56]. These discrepancies have been attributed to possible differences in experimental methods, endometriosis subtypes and cell types analyzed, and resolution of PGR isoforms [57]. It is also attributble to “patchy” endometrium [58], i.e., endometrial heterogeneity within the same patient. 

However, reduced PGR expression, or even PR-B promoter hypermethylation in the endometrium is not exclusively confined to patients with endometriosis. Endometrial PR-B expression also is reduced in patients with adenomyosis [59], and its reduction may also be attributed to its promoter hypermethylation [44]. In poorly differentiated endometrial cancer, PR-B expression is reduced as well [60]. In women wearing a levonorgestrel-releasing intrauterine system (LNG-IUS), total PGR and PR-B staining in the endometrium are significantly reduced [61]. Therefore, reduced endometrial PGR or PR-B expression is not a sufficient condition for endometriosis. This scenario, called “one-to-many”, is depicted in Figure 1B. 

The last example is promoter hypermethylation of HOXA10 in eutopic endometrium from women with endometriosis. First reported in 2005 [62], it was later independently confirmed in baboon and mouse models of endometriosis [63,64], as well as in humans [65,66]. The silencing of endometrial HOXA10 because of promoter hypermethylation is associated with decreased fertility, implantation defects and/or the reproductive wastage seen in certain disease states that affect the female reproductive tract [67]. However, promoter hypermethylation of HOXA10 in the endometrium turns out to occur not only in patients with endometriosis but also those with Asherman’s syndrome, intramural and submucosal uterine myoma, as well as endometrial polyps [67]. The endometrium of women wearing intrauterine devices also carry HOXA10 methylation [68]. Therefore, again, this molecular aberration is not exclusively confined to patients with endometriosis, and, as such, does not qualify as a sufficient condition (Figure 1C).

Through the above three examples, we can conclude that many molecular/cellular aberrations in the endometrium of women with endometriosis are either not universal to all patients with endometriosis (as in endometrial hyperinnervation restricted to those complaining of pain), or not exclusively confined to those with endometriosis. In other words, these aberrations are not a sufficient condition, and in some cases not even a necessary condition. Most importantly, however, since these aberrations are found, post hoc, in the endometrium from women who have been already diagnosed with endometriosis, there is no way to tell whether these aberrations are the cause, or merely the consequences, of endometriosis. Of course, for a complex disease such as endometriosis, a linear cause-and-effect relationship seldom exists during the entire course of the disease progression. Many factors could initially be the consequence of the disease but later become part of the causal complex. However, to truly qualify for “endometrial determinism”, the endometrial aberration must occur first, endowing the aberration-carrying menstrual debris the ability to invade, adhere to ectopic sites and then evade immune surveillance, detection and clearance, and cause symptoms. That is, it predisposes its bearer to endometriosis. Therefore, two additional requirements are needed to establish “endometrial determinism” (Figure 1C,D). In scenario C (the consequence), the endometrial aberration is the consequence of endometriosis, and the aberration may vary depending on the subtype of endometriotic lesion and its proximity to the uterus, as reviewed below. In scenario D (the third party), both endometrial aberration and endometriosis could be caused by a third party, which could be linked to in utero exposure of high levels of estrogens [69,70] or some inborn errors or other extraneous factors yet to be identified. 

When exploring the hypothesis that endometriosis originates from the endometrium harboring molecular aberrations, it should be considered that differences between ectopic and the eutopic endometrium could be secondary to the vast differences in the microenvironment existing between the two locations. The ectopic endometrium in endometriosis is influenced by the peritoneal fluid, or by local microenvironments in the case of ovarian endometrioma [71]. In turn, ectopic endometrium could influence gene expression in the eutopic endometrium, since endometrial tissue implanted at sites proximal and distal to the uterus in the mouse model of endometriosis alters gene expression in the eutopic endometrium. Furthermore, the changes in gene expression differ in relation to the site of implantation [72]. 

In addition to aberrations in eutopic endometrium, emerging data also indicate that endometriosis may cause systemic changes. In mice with induced endometriosis, dysregulated hepatic metabolic genes have been demonstrated [73]. This may be responsible for the low body mass index observed in women with endometriosis [74]. Recently, it has been reported that endometriosis promotes atherosclerosis in mice [75]. 

## 3. Aberrations in the Eutopic Endometrium Are Secondary to the Establishment of Ectopic Foci

All existing evidence that eutopic endometrium from women with endometriosis harbor certain molecular aberrations came, without exception, from patients who have already been diagnosed with endometriosis. In other words, the aberrations found in eutopic endometrium are identified post hoc in women who have already been diagnosed with the disease. Although on the surface this may be consistent with the notion that endometriosis originates from defective endometrium, it cannot be taken as de facto evidence. In fact, it goes without saying that to prove the causal relationship, a temporal, as well as a phylogenetic, relationship needs to be firmly established between eutopic endometrium and endometriotic lesions. 

Although it is ethically challenging as well as logistically difficult to follow up women who may or may not have aberration-bearing endometrium, consistent data from animal experimentations demonstrate that eutopic endometrium actually acquires molecular aberrations after the induction of endometriosis [63,64,72,76,77]. 

In baboon models of endometriosis, a significantly reduced endometrial expression of HOXA10 and integrin β3 (ITGB3), which are known to be involved in endometrial receptivity [78,79,80], has been reported one year after the induction of endometriosis [63]. In particular, the promoter region of the HOXA10 gene is hypermethylated, rendering it silent [63]. In this model, endometriosis was induced by intraperitoneal inoculation with menstrual endometrium on day 2 of two consecutive menstrual cycles [81]. No endometrial aberration has ever been reported in baboons except after induction of endometriosis. Therefore, it can be concluded that the molecular aberrations seen in eutopic endometrium occurred after the induction of endometriosis. 

It has also been demonstrated that endometrial response to hCG, in the same baboon model, is altered after induction of endometriosis. Furthermore, the aberrant response of the eutopic endometrium varies with disease progression [77]. In mice with apparently normal endometrium, endometrial expression of Hoxa10, Hoxa11 and Igfbp1 (Insulin-like growth factor binding protein-1) was significantly reduced 14 weeks after the induction of endometriosis [64]. Hoxa10 is reported to be hypermethylated in both the mouse and the baboon model [64].

Endometrial response also varies based apparently on lesion location. Endometrial expression of Hoxa10, Hoxa11, Igfbp1, Klf9 (Kruppel-like factor 9) and PGR was reduced in mice when endometriosis was induced in the peritoneal cavity (proximal) compared to control eutopic endometrium. The difference was statistically significant (*p* ≤ 0.04) [72]. In cases where endometriosis was implanted subcutaneously at distant sites (distal), endometrial gene expression was little affected except for Igfbp1 and PGR (*p* ≤ 0.02) [72]. This seems to suggest that endometrial aberrations in the mouse model of endometriosis are the consequence, rather than the cause, of endometriosis. It also demonstrates that endometriosis has systemic as well as recognized local effects. The effects of endometriosis on distant organs may also be responsible for some of its systemic manifestations [72].

It has been consistently reported that the endometrial expression of Hdac3 in mice with induced endometriosis is significantly reduced, resulting in increased fibrosis [76]. In addition, mice with induced endometriosis exhibit higher implantation failure [76]. 

Taken together, consistent experimental data have demonstrated, rather convincingly, that endometriosis is causally responsible for molecular aberrations in eutopic endometrium. This, of course, does not rule out that aberrations of the eutopic endometrium can act as triggers for the disease.

## 4. The Mandate to Establish a Phylogenetic and Causal Relationship 

Naturally, building a convincing theory of the origin of endometriosis requires that the eutopic endometrium can be demonstrated to harbor certain molecular aberrations that truly endow the menstrual debris, regurgitated into the pelvic cavity, with the ability to adhere, implant, invade ectopic locations and, equally importantly, evade immunosurveillance and defy removal. In addition, evidence should be produced to show, unequivocally, that the ectopic endometrium has indeed descended, by lineage, from an ancestor aberrant cell originally residing in eutopic endometrium. Just showing that both eutopic and ectopic endometrium share certain markers is not sufficient and far from conclusive. This is because although each endometriotic lesion is monoclonal [82], the endometrium is polyclonal [83,84]. Indeed, sequencing of the endometrial epithelium genome has shown that the epithelium has a heterogeneous rather than a homogenous structure [14]. Of course, when multiple lesions of possibly different subtypes exist within the same patient, there is a complex relationship among these lesions because of oligoclonality and metastasis [85].

**Figure 2:**
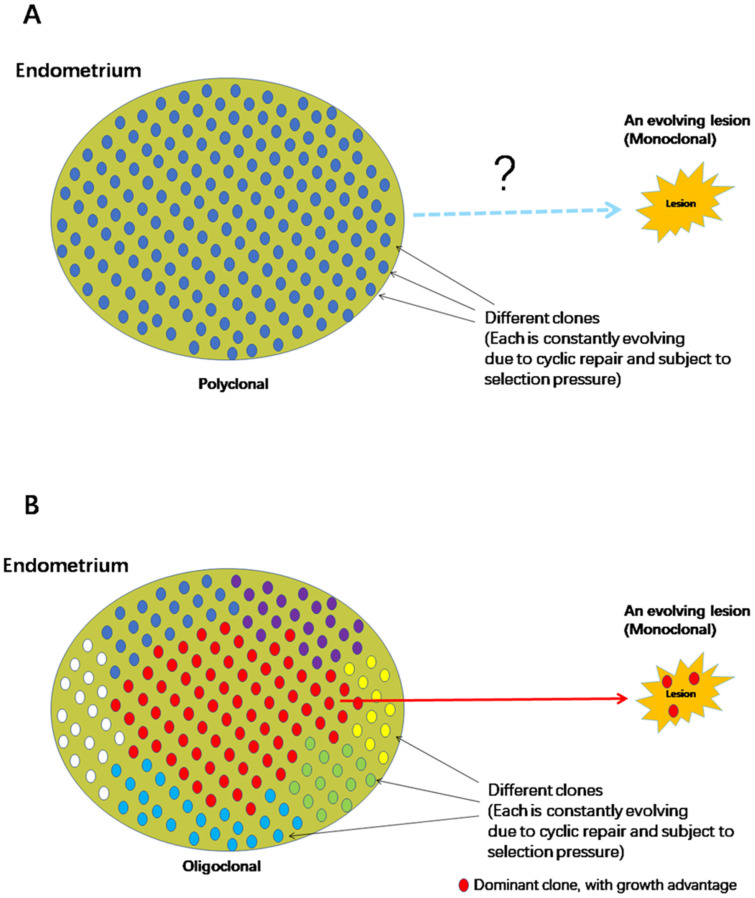
Two scenarios showing the complex phylogenetic relationship between the polyclonal endometrium and monoclonal endometriotic lesion. (**A**) Both eutopic and ectopic endometria are each evolving at their own pace and tempo and in their separate microenvironments, which makes any inference related to the phylogenetic relationship between the two entities challenging. (**B**) Because of cancer-associated mutations that confer selective growth advantages, the endometrium may become oligoclonal. Some clones may dominate and occupy larger areas of the endometrium. Since the chance of test sample coming from the dominant clone is thus increased substantially, this would help establish the phylogenetic relationship between eutopic and ectopic endometrium should the retrograde menstruation theory be true. However, it poses a challenge in establishing that a clone harbouring a particular mutation confers higher risk of developing endometriosis than those without.

As the number of clones that form the endometrium is supposedly not small, a random selection of any region for phylogenetic comparison cannot be considered to represent the whole endometrium and carries an inherent risk of error. As such, it is difficult to decide on the relevant region for comparison (Figure 2A). 

One possible silver-lining may be the presence of “rhizome structures” in the endometrium, in which the basal glands run horizontally along the muscular layer and multiple vertical glands, sharing the same somatic mutations rise from the basal gland, and originate from the same ancestral clone [86]. In this case, the number of clones with different CAMs may be reduced. 

However, this seemingly silver lining also raises the bar to establish the causal relationship between endometrial aberrations and the risk of developing endometriosis (Figure 2B). As CAMs are known to confer selective growth advantages to affected subclones as compared with surrounding cells [87,88,89], sooner or later some CAM-harboring clones would become larger and even become one of few dominant clones in the endometrium, as shown consistently in histologically healthy tissues [89,90]. Hence, chances are that a phylogenetic relationship can be established, linking endometriotic lesions with the dominant clone. However, this relationship may well be the result of selection for growth within the endometrium, not necessarily because the clone genuinely predisposes its owner to a higher risk of developing endometriosis. This is something akin to the well-known “survivor bias”. 

Second, as both the endometriotic lesion and the eutopic endometrium are constantly evolving by and on their own, it becomes even more difficult to demonstrate that any selected sample of eutopic endometrium and the lesion deposit share the same ancestral clone. Since all cells of a human body can be traced ultimately to a single ancestral cell, i.e., a fertilized egg, to convincingly demonstrate such a genealogic link, one needs to show, unequivocally, that: (1) the endometrial sample piece and the lesion are phylogenetically related; (2) they share a common ancestor that is not too distant; and (3) the lesional cells are the descendants of the sample piece. In other words, hard phylogenetic data is required to show that the lesion of interest is indeed descended from the clone that resides in the eutopic endometrium. Merely sharing similar markers cannot be taken as iron-clad evidence.

Lastly and most importantly, there is a mandate to prove that the endometrium carrying certain molecular aberrations will indeed substantially and meaningfully increase the risk of developing endometriosis. Given that nearly all epidemiological and experimental data are consistent with Sampson’s retrograde menstruation theory, it is just a matter of time until, if retrograde menstruation does indeed cause endometriosis, the ectopic endometrium will be traced back in lineage to eutopic endometrium. To prove that endometriosis originates from aberration-carrying endometrium, one would have to show that endometrium carrying these aberrations confer a significantly higher risk of developing endometriosis than those that do not.

Take CAMs as an example. CAMs, also called driver mutations, or oncogenic or tumor suppressor mutations, are induced by exogenous factors (such as reactive oxygen species, ultraviolent light, or exposure to mutagens) as well as endogenous factors (such as replication errors or aging). They are known to confer selective growth advantages to affected subclones as compared with surrounding cells [87,88,89]. Of relevance, CAMs in histologically normal endometrium can occur in women as young as 21 years, without endometriosis [86]. That is, the endometrium harboring a specific CAM is not necessary nor sufficient to cause endometriosis. 

Of course, although it was reported nearly two decades ago that the KRAS mutation causes endometriosis in mice and the KRAS mutation is indeed found in endometriotic lesions [14,85,91], there are plenty of data also showing that women with endometrium harboring KRAS mutations are apparently normal, i. e., without endometriosis [86,92,93]. More interestingly, in colorectal cancer, it has been recently found that a large proportion of the observed sub-clonal gene expression variations is not determined by heritable (i.e., genetic) variations through tumor evolution, suggesting that phenotypic plasticity—the ability of a cancer cell to change phenotype without underlying heritable (epi)genetic change—is a common phenomenon [94]. We do not know at this moment if this also happens in endometriosis, but if it does, then it would mean that different women whose endometria are carrying the same CAM may not all develop endometriosis. Therefore, aside from the phylogenetic relationship between eutopic and ectopic endometrium, additional evidence for the increased risk of endometriosis caused precisely by these endometrial aberrations needs to be established. 

Unfortunately, the data published so far only demonstrated that both eutopic and ectopic endometria in the same patient share similar CAMs [14], but no phylogenetic relationship has been established among these mutations. The latest research also shows that multiple lesions within the same patient share similar CAMs, demonstrating oligoclonality and metastasis of endometriotic lesions [85]. Yet so far, there has been no phylogenic evidence. Indeed, given the ubiquity of CAMs in both histologically normal tissues and the endometrium [92,95], the mere demonstration of the existence of CAMs in both eutopic and ectopic endometrium cannot be taken as evidence that endometriosis originates from endometrium that harbors molecular aberrations. Indeed, CAMs are not confined to endometriotic or adenomyotic lesions, but are present in other gynecological lesions, such as endometrial polyps that carry CAMs, such as KRAS [96]. Hence, just as endometrial hyperinnervation, or downregulation of PGR, or HOXA10 promoter hypermethylation, CAM-carrying endometria are not exclusively found in women with endometriosis, these aberrations are not sufficient to inevitably cause endometriosis exclusively. 

In theory, it is technically plausible to identify whether a phylogenetic relationship exists between eutopic and ectopic endometrium. As next generation sequencing (NGS) methods become increasingly available and affordable, it will be soon feasible to interrogate the phylogenetic relationship, if any, between or among different tissues in the same patient based on the molecular clock [97]. Recent methodological advancements, such as the use of the epimutations as a molecular clock [98] should be able to help determine whether there is any phylogenetic relationship between eutopic endometrium and endometriotic lesions.

However, to demonstrate the causal relationship would also require experimental data based on endometriosis models on animals that recapitulate the human condition, and even more prospective epidemiological studies. 

## 5. The “Root Treatment”: Practicality and Biological Plausibility Concerns

“Root treatment” or having the endometriotic lesion nipped in the bud may be the loftiest end-goal for endometriosis research, but this should demand more rigorous and careful research and data. When scrutinized closely, it would be easy to see that all endometrial aberrations in endometriosis reported so far are not sufficient nor necessary to cause endometriosis. Yet in medicine, the first prerequisite for a treatment or medical intervention is correct diagnosis. Can we diagnose endometrial aberrations that predispose women to endometriosis? 

The short answer is a resounding no. In fact, as alluded to above, any evidence-based diagnostic criteria or procedures for the identification of “defective endometrium” are not going to be available any time soon. Consequently, “root treatment” is nothing but a pipedream as of now. 

To establish that endometriosis originates from endometrial aberrations, the aberration, genetic, proteomic, or otherwise, needs to be clearly defined and detectable and verifiable by current biological or physical means, and should be proven to increase the risk of developing endometriosis in its bearers as compared with those who do not carry it. Ideally, an associated risk estimate, likely to be age- and time-dependent, should be provided. 

Naturally, to institute a “root treatment” to mitigate the risk of developing endometriosis, one essential prerequisite is the ability to identify or diagnose, with enough accuracy, the purported aberrations that predispose their owners to increased risk of endometriosis. Additionally, the other requirements are safety, efficacy, and desirable risk/benefit and cost/benefit ratios. Unfortunately, in the absence of any solid evidence indicating that endometriosis originates from the endometrium with molecular aberrations, as well as data on the safety and efficacy of such interventions, as of now the idea of “root treatment” is just a wishful thinking. 

There are numerous concerns. For the sake of argument, let us say that in the end a phylogenetic relationship between molecular aberrations in the eutopic and ectopic endometrium can be unequivocally established, demonstrating that, undisputedly, endometrial aberrations antedate the same aberrations in endometriotic lesions and, equally importantly, that there is a genealogical relationship linking these events. Would “root treatment” be practical or even biologically plausible? The Chinese consensus [13] is predictably evasive and utterly noncommittal in this regard. 

First, since the endometrium is polyclonal [83,84] and likely “patchy” [58], it is not a universally homogeneous tissue. Hence, sampling would pose the first challenge as to which part of endometrium to sample. Clearly, one cannot sample all endometria, and this being the case, the false negative rate is likely to be high. 

Second, CAMs in histologically normal endometrium are quite common [86,92,93], and many women with CAM-harboring endometrium are apparently normal. In fact, the events of CAMs and subsequent clonal expansions, as well as copy neutral loss-of-heterozygosity can occur early in life, suggesting such events can be tolerated for many years in normal endometrium. 

The majority of people harboring a lot of clonal CAMs in their tissues are still healthy without any apparent pathology. In fact, these CAMs merely confer selective advantages without exhibiting any malignancy or pathology [58,88,89,90,99]. Hence, is it still wise to intervene if an aberrant, CAM-harboring endometrium is detected? What is the chance that it would result in endometriosis in the next 5 years? Furthermore, if no aberration is found, how often should this screening be performed?

Third, since CAMs are DNA sequence changes, there is no drug that reverses or rectify these sequences. The best one could do is either to use inhibitors if there is an activating mutation, or to use agonists if there is an inactivating mutation, assuming that these agonists/inhibitors are available and have acceptable risk/benefit and cost/benefit ratios. Now, since in most cases these CAMs only confer growth advantages over non-CAM carrying clones, without causing overt malignancy or even pathology, how should one justify the ensuing intervention? How can we be sure that the intervention would not interfere with normal endometrial physiology and/or pregnancy? 

Lastly, even if such as diagnostic approach is available, how accurate is it? If a women is identified by this diagnostic procedure as a “at-risk” person, is there any prophylactic or interventional measure? Would the positive identification cause fear or anxiety, which by and in themselves could accelerate lesional development? 

Realistically, the catalogue and assessment of all CAMs in the endometrium that may increase the risk of developing endometriosis would take years, if not decades. The development of safe and cost-beneficial intervention would take additional time. As of now, given that there is no unequivocal evidence indicating that endometriosis originates from aberrant endometrium, the lack of any reliable detection method and of an intervention approach, it is safe to say that the “root treatment” is premature at best at this time. 

## 6. Conclusions

In light of the above discussion, we can see that although much evidence supports Sampson’s retrograde menstruation theory, an unequivocal genetic/molecular evidence of the *primum movens* of endometriosis is lacking simply because of the absence of phylogenetic data linking lesions to eutopic endometrium. This may change soon. However, to establish that it is indeed the endometrial aberrations that increase the risk of developing endometriosis to their bearers, the bar is much higher since this would not only require phylogenetic evidence, but also evidence to show that they do predispose their owners to endometriosis, after ruling out several alternative scenarios outlined herein. 

In many ways, to establish that endometriosis originates from endometrial aberrations is much harder and more time-consuming than developing biomarkers for diagnostic purposes. This would first require the establishment of a molecular “cartography of normal endometrium” that catalogues most, if not all, endometrial aberrations from the perspective of genomics, epigenomics, methylome, transcriptome, and/or proteomics for women of all age groups, which is an enormously daunting task. In addition, the recent report [92] that apparently healthy women in their teens could have CAMs in their endometrium also poses the question as what should be regarded as normal. It is also possible that one particular CAM may predispose its bearer to one particular disease but not to the other. Moreover, although the emerging single-cell genomic/epigenomic/transcriptomic/proteomic approaches may be very powerful in uncovering different cell populations and possibly different aberrations, proof of causality demands the establishment of a temporal relationship, which could take decades of painstaking follow-up. Of course, animal experimentations may help, but apart from higher primates, menstruation is quite rare in the animal kingdom, and the use of higher primates itself would pose enormous demand for resources. These challenges may explain why so far all efforts in such endeavors have been proven to be futile [26,100,101,102,103]. To show that one can do this and can also institute a “root treatment” is even more challenging, despite its lofty goal. Given all available data so far, that endometriosis results from retrograde menstruation is highly likely, but the chance that it originates from endometrium carrying certain molecular aberrations is yet unconvincing. “Root treatment” sounds lofty but as of now it is close to a fanciful Utopia. 

## Data Availability

Not applicable.
